# Development and Interpretation of a Genomic Instability Derived lncRNAs Based Risk Signature as a Predictor of Prognosis for Clear Cell Renal Cell Carcinoma Patients

**DOI:** 10.3389/fonc.2021.678253

**Published:** 2021-05-21

**Authors:** Huiying Yang, Xiaoling Xiong, Hua Li

**Affiliations:** Department of Nephrology, Sir Run Run Shaw Hospital, Zhejiang University School of Medicine, Hangzhou, China

**Keywords:** clear cell renal cell carcinoma (ccRCC), genomic instability (GI), somatic mutation profile, long non-coding RNA (IncRNA), risk signature, prognosis predicting, therapeutic scheme deciding

## Abstract

**Background:**

Clear cell renal cell carcinoma (ccRCC) is a kind of frequently diagnosed cancer, leading to high death rate in patients. Genomic instability (GI) is regarded as playing indispensable roles in tumorigenesis and impacting the prognosis of patients. The aberrant regulation of long non-coding RNAs (lncRNAs) is a main cause of GI. We combined the somatic mutation profiles and expression profiles to identify GI derived lncRNAs (GID-lncRNAs) in ccRCC and developed a GID-lncRNAs based risk signature for prognosis prediction and medication guidance.

**Methods:**

We decided cases with top 25% cumulative number of somatic mutations as genomically unstable (GU) group and last 25% as genomically stable (GS) group, and identified differentially expressed lncRNAs (GID-lncRNAs) between two groups. Then we developed the risk signature with all overall survival related GID-lncRNAs with least absolute shrinkage and selection operator (LASSO) Cox regression. The functions of the GID-lncRNAs were partly interpreted by enrichment analysis. We finally validated the effectiveness of the risk signature in prognosis prediction and medication guidance.

**Results:**

We developed a seven-lncRNAs (*LINC00460*, *AL139351.1*, *AC156455.1, AL035446.1*, *LINC02471*, *AC022509.2*, and *LINC01606*) risk signature and divided all samples into high-risk and low-risk groups. Patients in high-risk group were in more severe clinicopathologic status (higher tumor grade, pathological stage, T stage, and more metastasis) and were deemed to have less survival time and lower survival rate. The efficacy of prognosis prediction was validated by receiver operating characteristic analysis. Enrichment analysis revealed that the lncRNAs in the risk signature mainly participate in regulation of cell cycle, DNA replication, material metabolism, and other vital biological processes in the tumorigenesis of ccRCC. Moreover, the risk signature could help assess the possibility of response to precise treatments.

**Conclusion:**

Our study combined the somatic mutation profiles and the expression profiles of ccRCC for the first time and developed a GID-lncRNAs based risk signature for prognosis predicting and therapeutic scheme deciding. We validated the efficacy of the risk signature and partly interpreted the roles of the seven lncRNAs composing the risk signature in ccRCC. Our study provides novel insights into the roles of genomic instability derived lncRNAs in ccRCC.

## Introduction

Renal cell carcinoma (RCC) is one of the most common cancer types in urinary system, which originates from the renal epithelium and accounts for about 2% of all kinds of cancers diagnoses and deaths worldwide. The average annual incidence and death cases of RCC are about 295,000 and 134,000, respectively, and the incidence rate has been increasing over time (1). Clear cell renal cell carcinoma (ccRCC) is the main type of RCC and occupies about 80% - 90% of all RCC cases.

With the development of targeted therapy, immunotherapy, and other newly applied therapies, the clinical outcomes of a portion of patients have been improved. Whereas, according to the observations of genomic studies, there is overt molecular and cellular heterogeneity among ccRCC patients, which could contribute to the heterogeneous outcomes (2, 3). Thus, individualized evaluation and outcome prediction are in urgent need.

Genomic instability (GI) is a hallmark of most cancer, which arises from mutations and results in the occurrence of cancers (4). GI is a major cause of tumor heterogeneity within and between tumors (5). Moreover, it is broadly recognized that GI is closely related with the progression and prognosis of tumors (6–9).

Long non-coding RNA (lncRNA) is a kind of cellular transcripts that is larger than 200 nt and does not code for proteins, which mainly functions by transcription regulating, nuclear domain organizating, and proteins or RNA molecules regulating (10). Researchers have clarified that non-coding RNAs (ncRNAs) play indispensable roles in maintaining genomic stability as well as in the progression and invasion of cancers. Single nucleotide variants (SNVs) is the most frequently occurred mutation in tumors and most known cancer-related SNVs are related to the aberrant function of ncRNAs, especially lncRNAs (11, 12). Meanwhile, SNVs could regulate the expression level of corresponding ncRNAs in return (12).

The conception of GI is composed of three categories: instability of chromosome, chromatin higher-order structure, and DNA sequence (13). A series of research have proved that lncRNAs regulates the mitotic checkpoint and centromere proteins, and thus leads to aneuploidy formation (14–16). The up-regulation of telomeric repeat-containing RNA (a kind of telomeric lncRNA) contributes to the stabilization of shortened telomeres and results in chromosomal instability and tumorigenesis (17). The disruption of topologically associated domains (TADs) usually results in the abnormally activation and rearrangements of transcription, becoming an inducement of tumors (18). Normal functioning of lncRNAs participates in maintaining the stability of TADs (19, 20). DNA damage, especially DNA double-strand breaks (DSB) repair was closely related to tumorigenesis. In the processes of DSB repair, damage-induced lncRNA is regarded as a vital regulator in DSB misrepair, which would lead to GI and carcinogenesis (21, 22).

The heterogeneity of ccRCC is widely acknowledged, and the roles of GI in ccRCC have been broadly studied (23–26). While the role of GI-derived lncRNAs (GID-lncRNAs) in ccRCC has rarely been reported. It would be helpful to score tumors with GID-lncRNAs as the prognosis and therapeutic strategies may differ quite a lot from each other. In the present study, we attempted to integrate the expression profiles and somatic mutation profiles of ccRCC patients to construct a GID-lncRNA based risk signature for diagnosis prediction and therapies decision in ccRCC patients.

## Materials and Methods

### Study Design

**Figure 1:**
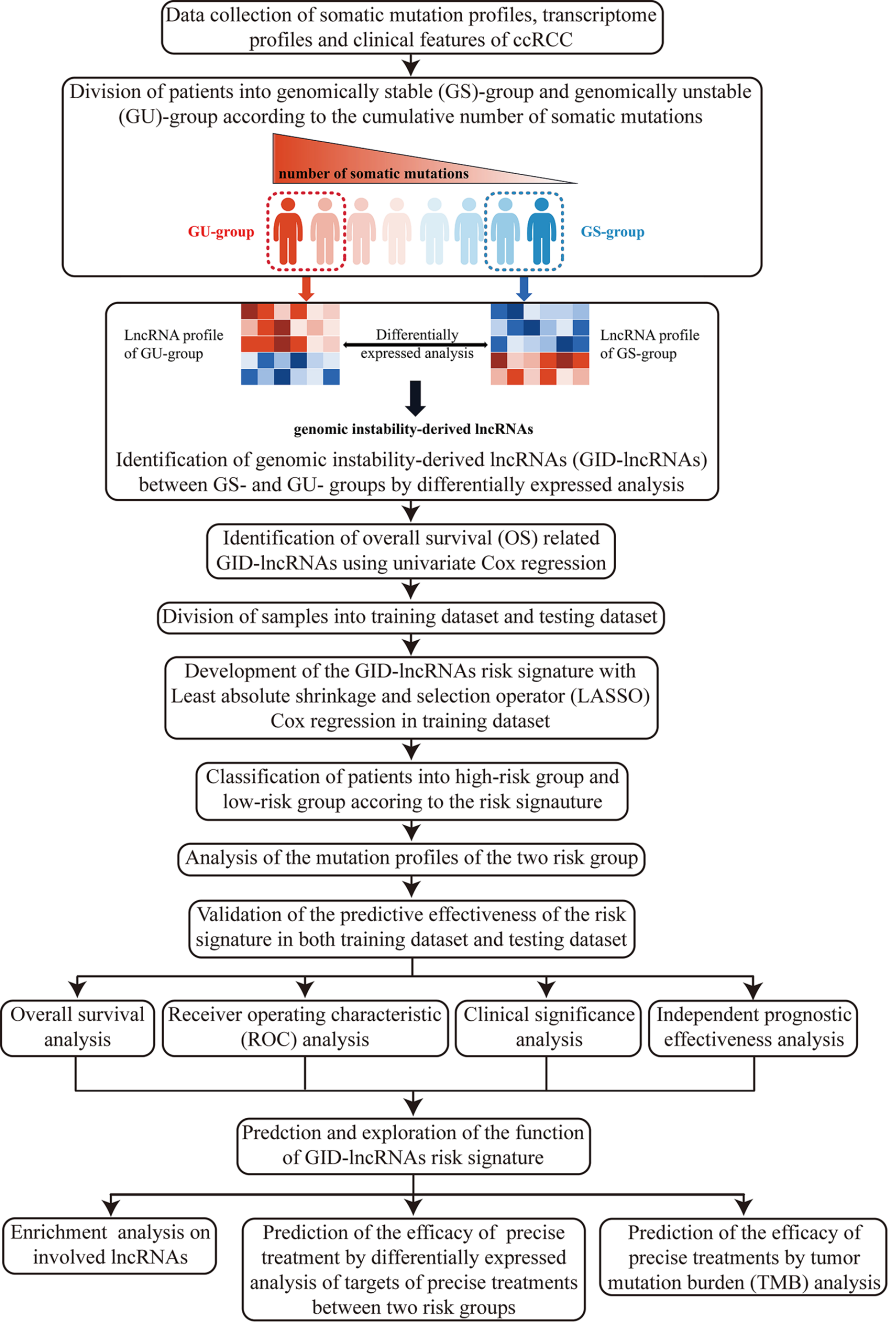
Flow chart of the design and overall procedures of our research.

Here, we firstly illustrated the overall design and procedures of the research (**Figure 1**).

### Data Collection

We collected the somatic mutation profiles, transcriptome profiles and clinical information of all ccRCC patients recorded in the database of The Cancer Genome Atlas Program (TCGA, https://portal.gdc.cancer.gov/) on 16th September, 2020. The research included the data of all 539 ccRCC tumor samples. The data of somatic mutations contains the cumulative number, mutation site, and mutation type in each sample. The data of transcriptome profile contains the expression level of detected RNAs in each sample. From the clinical information, we selected age, gender, follow-up time, survival status, tumor grade, pathological stage, TNM stage for analyses (information of lymphatic metastasis was not analyzed as it is missing in most cases and is not routinely assessed as an important criterion for prognosis predicting in ccRCC). As short follow-up period usually means inaccurate conclusions, we discarded the cases of follow-up period less than 3 months in cox regressions and survival analysis concerning follow-up time.

### Identification of the Genomic Instability Derived lncRNAs

The somatic mutation profiles of 336 cases were available and all cases were included in this part of analysis. Firstly, we calculated the cumulative number of somatic mutations for each patient. Then, we divided the patients into genomically stable (GS) group and genomically unstable (GU) group after ranking all patients according to the cumulative number of somatic mutations. Patients with top 25% numbers of somatic mutations were defined as GU group, and with last 25% numbers were defined as GS group. Finally, we performed differentially expressed (DE) analysis on all lncRNAs between the two groups. The analysis was conducted using Wilcoxon test with limma package (27) under R programming environment. The cut-off criteria for statistically significant difference were decided as false discovery rate (*FDR*) adjusted *p* < 0.05 and |log fold change (FC)| > 1. The identified DE-lncRNAs were regarded as GID-lncRNAs.

### Identification of Overall Survival Related GID-lncRNAs

Of all GID-lncRNAs, overall survival (OS) related genes usually perform critical functions in the onset and progression of tumors, as well as make decisive difference on prognosis. Thus, we performed time-dependent univariate Cox regression with survival R package to extract OS-related GID-lncRNAs. The cut-off criterion for statistically significant correlations with OS was decided as *p* < 0.05. After discarding cases with follow-up period less than 3 months, 491 cases were included in this part of analysis.

### Development of the GID-lncRNAs Based Risk Signature for Prognosis Prediction

The same 491 cases were included for constructing the risk signature. We divided all tumor cases into a training dataset and a testing dataset for developing and validating the risk signature. The cases were divided randomly according to a proportion of 7:3 (70% for training dataset and 30% for testing dataset, 377 cases in training dataset and 114 cases in testing dataset). We performed chi-square test to make sure that the training dataset and testing dataset were divided without bias.

Least absolute shrinkage and selection operator (LASSO) Cox regression was applied for develop a GID-lncRNAs based risk signature. The LASSO Cox regression would pick out variables for constructing the signature and give them coefficients. The GID-lncRNAs based risk signature was constructed as follow:

$$\begin{array}{c} \text{Risk}\;\text{score}=\text{expression}\;\text{level}\;\text{of}\;\text{lncRNA}1*\beta 1+\text{expression}\;\text{level}\;\text{of}\;\text{lncRNA}2*\beta 2+\\ \ldots +\text{expression}\;\text{level}\;\text{of}\;\text{lncRNAn}*\beta n,\;\text{where}\;\text{risk}\;\text{score}\;\text{is}\;\text{a}\;\text{measure}\;\text{for}\;\text{the}\\ \text{prognosis}\;\text{of}\;\text{ccRCC}\;\text{patients}\;\text{and}\;\beta \;\text{is}\;\text{the}\;\text{regression}\;\text{coefficient}\;\text{of}\;\text{each}\;\text{variable}. \end{array}$$

In both of the training dataset and the testing dataset, we computed the risk score for each patient according to the risk signature and then divided the patients into high-risk group and low-risk group from the median value of the risk score.

### Analysis of the Mutation Profiles of the Two Risk Groups

In order to assess the degree of GI and predict the prognostic outcomes of ccRCC patients with GID-lncRNAs, we have successfully developed the risk signature and divided patients into high-risk and low-risk groups. It is expected that patients in high-risk group are at GU condition. In order to confirm the supposition, we compared the mutation profiles of the two groups with maftools R package (28). All 336 cases with mutation profiles were included in this part.

### Verification of the Effectiveness in Prognosis Predicting of the Risk Signature

After the development of the risk signature, we have to assess its reliability and robustness in estimating the prognostic outcomes of patients. The effectiveness of the signature was validated from the following 4 aspects:

- Overall survival analysis was conducted in training and testing datasets respectively to explore if the survival rate or survival time was significantly different between high-risk and low-risk groups. We conducted survival analysis using Kaplan-Meier method with a two-sided log-rank test (with survival R package). 377 cases were included in training dataset and 114 cases were included in testing dataset.
- Receiver operating characteristic (ROC) analysis was conducted in training and testing datasets respectively to evaluate the efficiency of the risk signature in predicting the survival status using survivalROC R package. 377 cases were included in training dataset and 114 cases were included in testing dataset.
- Inter-group differences of clinicopathologic features were analyzed with chi-square test for exploring whether higher risk score is corresponding to more severe clinicopathologic status. After excluding cases included missing data in tumor grade, pathological stage, or TNM stage, 457 cases were included.
- Univariate and multivariate Cox regression analyses were performed to assess if the risk signature could work as independent prognostic predictor of survival in ccRCC patients. 491 cases were included.

### Function Predicting of lncRNAs Included in the Risk Signature With Enrichment Analysis

LncRNAs do not code proteins themselves, but work through regulating protein-coding genes. In order to explore the potential function of the lncRNAs involved in the risk signature in ccRCC, we calculated the Pearson’s correlation coefficients between the lncRNAs and all mRNAs in the transcriptome profile to evaluate the co-expression relationships between lncRNAs and mRNAs. Then we extracted the top-300 related mRNAs for each lncRNAs and conducted Kyoto Encyclopedia of Genes and Genomes (KEGG) (29) enrichment analysis on these related mRNAs, respectively. The cut-off criterion for significantly enriched items was decided as *p* < 0.05. The analysis was conducted using clusterProfiler R package (30).

### Efficacy Prediction of Precise Treatments With the Risk Signature

In addition of predicting the prognostic outcome of ccRCC patients, the risk signature would be more serviceable if it could provide guidance for deciding treatment selections. With the development of precision medicine, ccRCC patients are given more opportunities and choices for curing or improving outcomes. We selected several targets of immunotherapy and targeted therapy approved by Food and Drug Administration (FDA) for analysis. The included targets of precise treatment are listed as follow: *mTOR*, *KIT*, *PD-1*, *PD-L1*, *PDGFRA*, *PDGFRB*, *VEGFR1*, *VEGFR3*, *FLT3*, *RET*, *MET*. We applied differentially expressed analysis for these targets between high-risk group and low-risk group to explore whether the expression level of these targets for precise treatments were related to different risk groups. The criteria for DE-genes were set as *FDR* adjusted *p* < 0.05 and |logFC| > 1.

### Efficacy Prediction of Immunotherapy With the Risk Signature Using Tumor Mutation Burden Analysis

Immunotherapy is a breakthrough for the treatment of ccRCC, but the overall response rate of *PD-1*/*PD-L1* inhibitors is not satisfactory. It is certified that tumor mutation burden (TMB) could effectively predict the efficacy of immunotherapy of tumors as a biomarker (31). We calculated the TMB value (mutations per million bases) for each patient and compared the TMB values between high-risk and low-risk groups by t-test to explore whether the risk signature could help predict the response to immunotherapies targeting *PD-1*/*PD-L1*.

## Results

### Identification of GID-lncRNAs With Differentially Expressed Analysis

**Figure 2:**
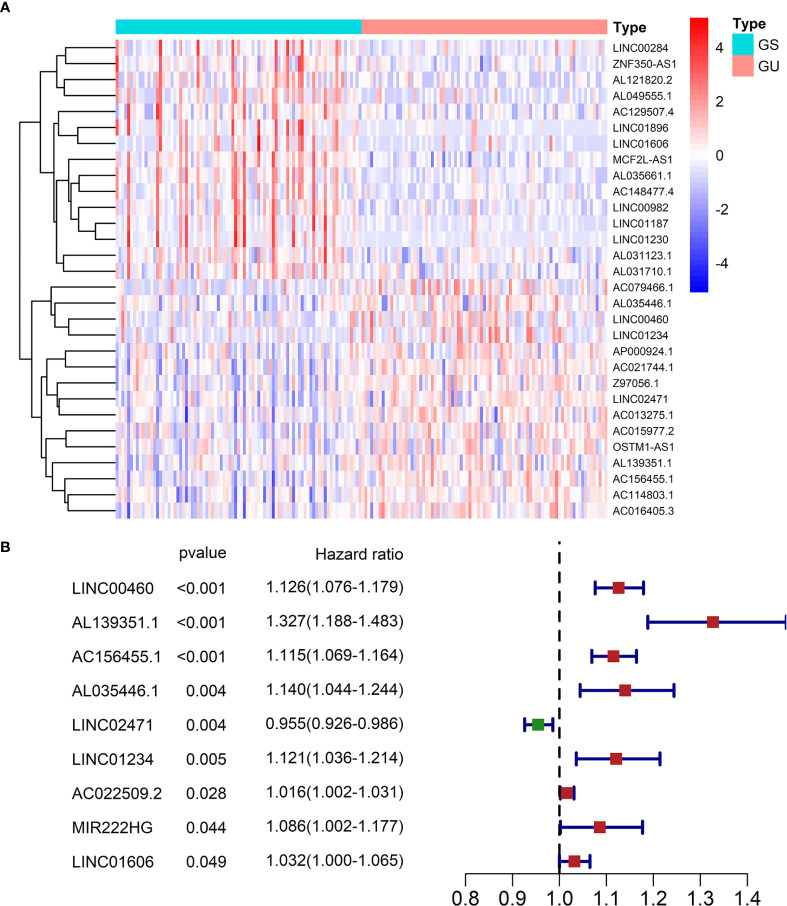
**(A)** A heatmap of all GID-lncRNAs between GS-group and GU-group. Each cell represents the expression level of a lncRNA (left) in a sample (above). Red means high expression and blue means low expression. The expression values were log2 transferred before mapping. **(B)** OS-related GID-lncRNAs recognized by time-dependent univariate Cox regression.

After computing the number of somatic mutations, patients were divided into GS-group and GU-group (The two groups contained 85 patients respectively, the ID and mutation number of patients are available in **Table S1**). We then performed DE analysis on all lncRNAs between GS-group and GU-group. We identified 46 DE-lncRNAs in all, which were regarded as functioning in GI (that is, GID-lncRNAs). Sixteen GID-lncRNAs were up-regulated in GU-group and 30 GID-lncRNAs were down-regulated in GU-group. The expression profiles of the GID-lncRNAs were shown in **Figure 2A** and the details (expression level, logFC, *p* value, *FDR* value) were given in **Table S2**.

### Identification of OS-Related GID-lncRNAs

Time-dependent univariate Cox regression recognized 9 OS-related lncRNAs from all 46 GID-lncRNAs (**Figure 2B**). The official gene names of the 9 OS-related GID-lncRNAs were *LINC00460*, *AL139351.1*, *AC156455.1*, *AL035446.1*, *LINC02471*, *LINC01234*, *AC022509.2*, *MIR222HG*, and *LINC01606*.

### Development of the GID-lncRNAs Based Risk Signature

Before developing the risk signature, we have divided all samples (with transcriptome profiles, follow-up time, and survival status) into training dataset and testing dataset. Chi-square test confirmed that there is no significant difference in clinical features between the two datasets. The information of patients in the two datasets and results of chi-square test are shown in **Table 1**.

**Table 1:** Clinical information of patients in training and testing dataset and chi-square test between two groups.

Covariates		Total	Training dataset	Testing dataset	p-value
Survival status, no (%)	Alive	340(69.25)	258(68.44)	82(71.93)	0.55
	Dead	151(30.75)	119(31.56)	32(28.07)	
Age, no (%)	<=65	328(66.8)	251(66.58)	77(67.54)	0.94
	>65	163(33.2)	126(33.42)	37(32.46)	
Gender, no(%)	Female	168(34.22)	133(35.28)	35(30.7)	0.43
	Male	323(65.78)	244(64.72)	79(69.3)	
Grade, no(%)	G1	10(2.04)	9(2.39)	1(0.88)	0.73
	G2	212(43.18)	159(42.18)	53(46.49)	
	G3	196(39.92)	150(39.79)	46(40.35)	
	G4	67(13.65)	54(14.32)	13(11.4)	
	Gx	6(1.22)	5(1.33)	1(0.88)	
Stage, no(%)	Stage I	246(50.1)	188(49.87)	58(50.88)	0.45
	Stage II	53(10.79)	39(10.34)	14(12.28)	
	Stage III	111(22.61)	82(21.75)	29(25.44)	
	Stage IV	78(15.89)	66(17.51)	12(10.53)	
	Stage x	3(0.61)	2(0.53)	1(0.88)	
T group, no(%)	T1	252(51.32)	193(51.19)	59(51.75)	0.65
	T2	65(13.24)	47(12.47)	18(15.79)	
	T3	165(33.6)	129(34.22)	36(31.58)	
	T4	9(1.83)	8(2.12)	1(0.88)	
Metastasis, no(%)	M0	390(79.43)	296(78.51)	94(82.46)	0.08
	M1	75(15.27)	64(16.98)	11(9.65)	
	Mx	26(5.3)	17(4.51)	9(7.89)	

We developed the risk signature in the training dataset. All 9 OS-related lncRNAs were involved in LASSO Cox regression to construct the risk signature for predicting the prognostic outcomes of ccRCC patients. While constructing the risk signature, LINC01234 and MIR222HG were eliminated by LASSO Cox regression.

The GID-lncRNA based risk signature was given as follow:

$$\begin{array}{l} \text{Risk}\;\text{score}\;=\;(0.0186884492327243\\ {}*\;\text{expression}\;\text{level}\;\text{of}\;L\text{I}NC00460)\\ +\;(0.163927492372108\;*\;\text{expression}\;\text{level}\;\text{of}\;AL139351.1)\\ +\;(0.145677628024267*\;\text{expression}\;\text{level}\;\text{of}\;AC156455.1)\\ +\;(0.0813180428494581\;*\;\text{expression}\;\text{level}\;\text{of}\;AL035446.1)\\ +\;(-0.0101523795313971\;*\;\text{expression}\;\text{level}\;\text{of}\;LINC02471)\\ +\;(0.00453936726148382\;*\;\text{expression}\;\text{level}\;\text{of}\;AC022509.2)\\ +\;(0.019535978614558\;*\;\text{expression}\;\text{level}\;\text{of}\;LINC01606) \end{array}$$

The detailed information of the 7 lncRNAs was provided in **Table 2**. Of the 7 lncRNAs composing the risk signature, 6 have positive coefficients and one has a negative coefficient. The up-regulation of lncRNAs with positive coefficients means worse outcome, that is, these lncRNAs may function as risky factors in ccRCC patients. Oppositely, lncRNA with negative coefficient may act as a protective factor.

**Table 2:** Information of the seven GID-lncRNAs composing the risk signature.

Gene Symbol	Ensembl ID	Genomic location	Coefficient in the risk signature
LINC00460	ENSG00000233532	chr13:106,374,477-106,384,411	0.018688449
AL139351.1	ENSG00000276923	chr20:48,024,788-48,074,227	0.163927492
AC156455.1	ENSG00000256546	chr12:122,063,306-122,068,616	0.145677628
AL035446.1	ENSG00000234147	chr6:140,807,603-140,898,430	0.081318043
LINC02471	ENSG00000223914	chr12:40,155,757-40,211,419	-0.01015238
AC022509.2	ENSG00000256234	chr12:26,211,164-26,335,856	0.004539367
LINC01606	ENSG00000253301	chr8:57,142,659-57,244,924	0.019535979

For the subsequent analyses, we calculated the risk score for each patient with our risk signature and ranked patients according to their risk scores in training dataset and testing dataset, respectively. Then, patients were classified into high-risk group and low-risk group according to the median value of the risk score as a threshold (0.321 in training dataset and 0.329 in testing dataset). Samples in training dataset and testing dataset, their expression profile of the seven lncRNAs, as well as their risk scores and risk groups were given in **Table S3**.

### Analysis of the Mutation Profiles of the Two Risk Groups

**Figure 3:**
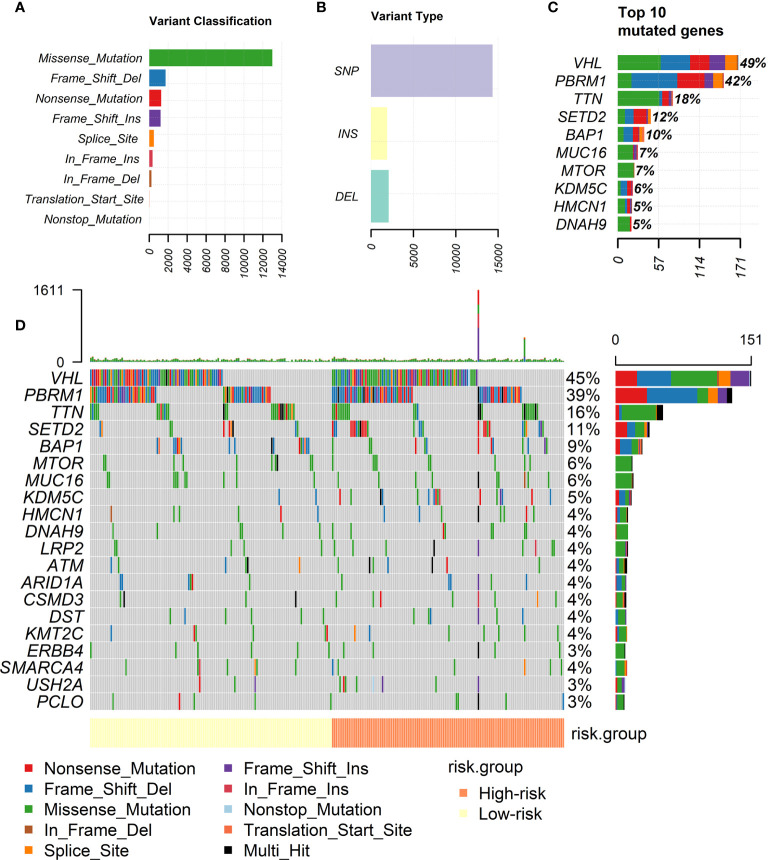
Landscape of mutation profiles of ccRCC patients. **(A)** Classification of mutations by their effects. **(B)** Classification of mutations by different patterns. **(C)** Top 10 frequent mutations in ccRCC patients. **(D)** Mutation profiles of high-risk and low-risk groups divided by our risk signature.

**Figures 3A–C** showed the landscape of the mutation profiles of ccRCC patients. According to different classified categories, missense mutation is the most frequently occurred type of mutations in ccRCC, and the amount of single nucleotide polymorphism (SNP) is significantly larger than that of insertion (INS) or deletion (DEL). VHL is the most frequently mutated gene in ccRCC, occupying almost 50% of all patients.

**Figure 3D** showed the mutation profiles of high-risk patients and low-risk patients separately. Mutations were more frequently occurred in high-risk group. Meanwhile, the mutation of several genes is rarely observed in low-risk group but frequently observed in high-risk group, for example, *KDM5C*. More types of mutations were observed in high-risk group. The analysis intuitively reflected the differences of mutation profiles between high-risk and low-risk groups.

### Verification of the Effectiveness of Prognosis Prediction of the Risk Signature

**Figure 4:**
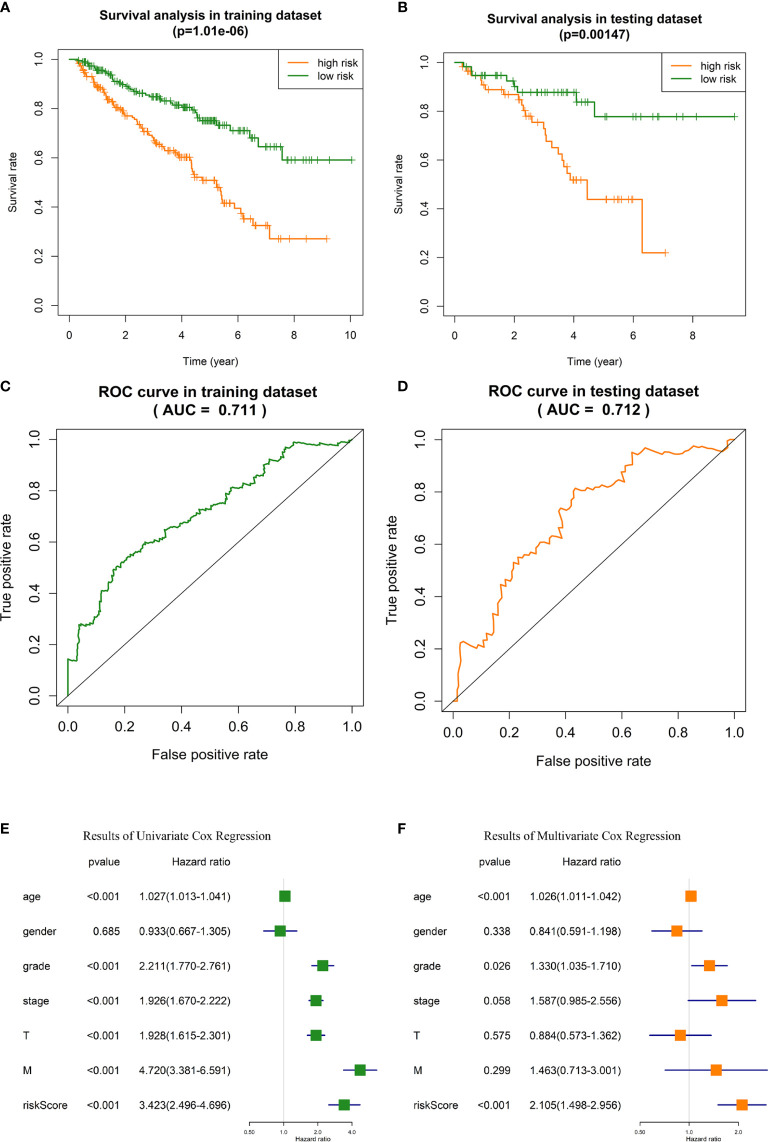
Validation of the GID-lncRNAs based risk signature. **(A)** Survival analysis in training dataset. **(B)** Survival analysis in testing dataset. **(C)** ROC analysis for evaluating the predictive efficiency of the risk signature in the training dataset. **(D)** ROC analysis for evaluating the predictive efficiency of the risk signature in the testing dataset. **(E)** Results of Univariate Cox regression. **(F)** Results of Multivariate Cox regression.

- OS analysis in training dataset and testing dataset indicated that the outcome of high-risk ccRCC patients identified with our risk signature was significantly poorer than low-risk patients (*p* = 1.01E-6 and 0.001, respectively, **Figures 4A**, **B**). In training dataset, the 5-year survival rate of patients was about 48% in high-risk group, and 74% in low-risk group. In testing dataset, the 5-year survival rate was 43% and 78%, respectively.
- In ROC analysis, the areas under the curve (AUC) of training dataset and testing dataset were 71.1% and 71.3%, respectively (**Figures 4C, D**), indicating the satisfactory robustness of the risk signature in prognosis predicting.
- Chi-square test between high-risk and low-risk groups validated that the portion of patients with higher tumor grade, pathological stage, T stage, and distant metastasis were significantly higher in high-risk group than that of low-risk group (**Table 3**, the *p* values were 4.27E-8, 2.41E-10, 1.00E-8, and 1.81E-4, respectively).
- Univariate and multivariate Cox regression analyses identified the risk signature as an independent risk factor for ccRCC patients (**Figures 4E, F**), which means the risk score calculated with our risk signature could predict the outcomes of ccRCC patients independent of the clinicopathologic features of tumor grade, pathological stage, T stage, and distant metastasis.

**Table 3:** Clinical features of patients in high-risk and low-risk groups and chi-square test between two groups.

Covariates		Total	High-risk	Low-risk	p-value
Survival status, no (%)	Alive	310(67.83)	126(55.51)	184(80.00)	3.70E-8
	Dead	147(32.17)	101(44.49)	46(20.00)	
Age, no (%)	<=65	303(66.3)	145(63.60)	158(69.00)	0.23
	>65	154(33.7)	83(36.40)	71(31.00)	
Gender, no(%)	Female	153 (33.5)	48(21.15)	105(50.7)	5.00E-8
	Male	304(66.5)	179(78.85)	125(49.3)	
Grade, no(%)	G1	7(1.53)	1(0.44)	6(2.61)	4.27E-8
	G2	198(43.33)	75(33.04)	123(53.48)	
	G3	187(40.92)	100(44.05)	87(37.83)	
	G4	65(14.22)	51(22.47)	14(6.09)	
Stage, no(%)	Stage I	225(49.23)	80(35.23)	145(63.04)	2.41E-10
	Stage II	47(10.28)	19(8.37)	28(12.17)	
	Stage III	109(23.85)	75(33.04)	34(14.78)	
	Stage IV	76(16.63)	53(23.35)	23(10.00)	
T group, no(%)	T1	231(50.55)	84(37.00)	147(63.91)	1.00E-8
	T2	58(12.69)	28(12.33)	30(13.04)	
	T3	159(34.79)	109(48.02)	50(21.74)	
	T4	9(1.97)	6(2.64)	3(1.30)	
Metastasis, no(%)	M0	383(83.81)	175(77.09)	208(90.43)	1.81E-4
	M1	74(16.19)	52(22.91)	22(9.57)	

### Function Predicting of lncRNAs Included in the Risk Signature With Enrichment Analysis

**Figure 5:**
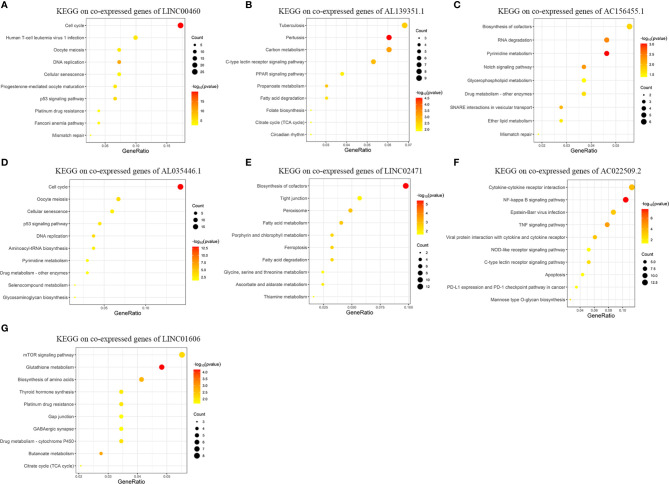
Results of KEGG enrichment analysis on genes co-expressed with GID-lncRNAs in the risk signature. The size of the dots means the count of genes enriched in the term and the color is corresponding to the statistical significance. Gene ratio means the ratio of genes enriched in the term and all genes involved in the analysis. **(A)** KEGG enrichment analysis on genes co-expressed with LINC00460. **(B)** KEGG enrichment analysis on genes co-expressed with AL139351.1. **(C)** KEGG enrichment analysis on genes co-expressed with AC156455.1. **(D)** KEGG enrichment analysis on genes co-expressed with AL035446.1. **(E)** KEGG enrichment analysis on genes co-expressed with LINC02471. **(F)** KEGG enrichment analysis on genes co-expressed with AC022509.2. **(G)** KEGG enrichment analysis on genes co-expressed with LINC01606.

**Figure 5** exhibits the top-10 enriched KEGG-terms of KEGG enrichment on the co-expressed genes of the seven lncRNAs in the risk signature (Details and all terms of the results of KEGG enrichment analysis are available in **Table S4**). *LINC00460* is closely related to signal pathways of “Cell cycle”, “DNA replication”, “*p53* signaling pathway”, and “Mismatch repair”. *AL139351.1* is involved in signal pathways concerning material metabolism and energy cycle. *AC156455.1* is related to “RNA degradation”, “Drug metabolism”, and “Mismatch repair”. *AL035446.1* takes part in signal pathways of “Cell cycel”, “*p53* signaling pathway”, “DNA replication”, and “Drug metabolism”. *LINC02471* mainly functions in signal pathways about material metabolism. *AC022509.2* is related to “*NF-kappa B* signaling pathway”, “*TNF* signaling pathway”, “Apoptosis”, and “*PD-L1* expression and *PD-1* checkpoint pathway in cancer”. *LINC01606* plays roles in “*mTOR* signaling pathway”, “Platinum drug resistance”, and “Drug metabolism”.

According to the results of KEGG enrichment analysis, we could conclude that the seven lncRNAs are involved in several biological themes closely related to the development and progression of tumors, such as cell cycle, DNA replication, and mismatch repair. Meanwhile, these lncRNAs may play important roles in material metabolism. Moreover, the functions of the lncRNAs have enriched in several widely admitted tumor-related signal pathways, such as *NF-kappa B* signaling pathway, *p53* signaling pathway, *mTOR* signaling pathway, and *TNF* signaling pathway. Most importantly, the aberrant regulation of these lncRNAs may contribute to the resistance towards chemotherapy and immunotherapy.

### Application of the Risk Signature in Predicting The Efficacy of Precise Treatments

**Figure 6:**
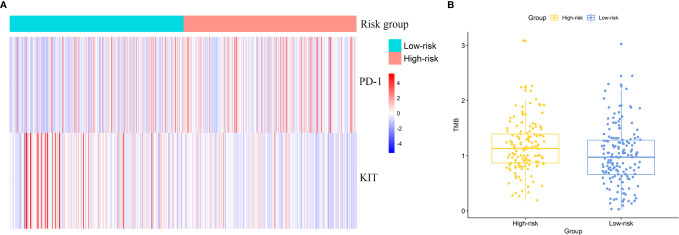
Effectiveness prediction of precise treatments with our risk signature. **(A)** Differentially expressed targets for precise treatments. **(B)** TMB analysis of ccRCC patients and significant differences between high-risk and low-risk group.

DE analysis found the significantly different expression level of *KIT* and *PD-1* between two risk groups (**Figure 6A**). *PD-1* was up-regulated in the high-risk group (logFC = 0.55, *FDR* = 2.17E-5) and *KIT* was down-regulated in the high-risk group (logFC = -1.92, *FDR* = 1.34E-5). The results hint that patients in high risk group might response better to treatments targeting *PD-1* and worse to treatments targeting *KIT*.

### Efficacy Prediction of Immunotherapy Using Tumor Mutation Burden Analysis

We calculated the TMB value for each patient and compared the inter-group difference of TMB value with t-test. The average TMB value in high-risk group was significantly higher than that in low-risk group. The TMB value was 1.25 in high-risk group and 1.00 in low-risk group (*p* = 0.019). **Figure 6B** exhibited the difference of TMB between two groups (one sample in high-risk group was omitted in the figure as its TMB value was too large). The results indicated a potential higher response rate towards immunotherapy in patients of the high-risk group classified by our risk signature.

## Discussions

In the present study, we integrated the analysis of the somatic mutation profiles and the transcriptome profiles of ccRCC patients to develop a GID-lncRNAs based risk signature for predicting the prognostic outcomes and deciding therapeutic strategies for ccRCC patients. We obtained the expression profiles, somatic mutation profiles, as well as clinical information of all ccRCC patients from TCGA database. We analyzed the somatic mutation profiles and classified patients into GS group and GU group. Differentially expressed analysis of transcriptome profiles between GS and GU groups identified GID-lncRNAs. Univariate Cox regression recognized 9 OS-related GID-lncRNAs and LASSO Cox regression developed a 7-lncRNAs risk signature for prognosis prediction. Using the risk signature, we classified all patients into high-risk and low-risk groups for subsequent validation and interpretation. Landscape of the mutation profiles showed the general information of somatic mutations in ccRCC patients and differences between the two risk groups. Survival analysis proved a worse prognosis in high-risk group and ROC analysis confirmed the satisfactory accuracy of prognosis predicting. Chi-square test between two risk groups validated that patients in high-risk group were in more severe clinicopathologic conditions. Cox regression proved the risk score calculated by our risk signature as an independent prognostic predictor for predicting the overall survival of ccRCC patients. KEGG enrichment analysis on co-expressed genes of the seven lncRNAs in the risk signature explored the potential role of these lncRNAs in ccRCC. The seven lncRNAs may play crucial roles in cell cycle, DNA replication, mismatch repair, and material metabolism in the development and progression of ccRCC. Finally, we attempted to predict the efficacy of precise treatments with our risk signature. DE analysis between the two groups found higher expression level of *PD-1* and low expression level of *KIT* in high-risk group, that is, patients in high-risk group might response better to treatments targeting *PD-1* and worse to treatments targeting *KIT*. TMB analysis detected a significant higher average TMB value in high-risk group, hinting a possibility of better response to immunotherapy targeting *PD-1* and *PD-L1* in high-risk group.

Up to now, the roles of lncRNAs in inducing GI in ccRCC have rarely been reported. We designed and performed the present study to screen out GID-lncRNAs in ccRCC and develop a GID-lncRNAs based risk signature for prognosis prediction and therapies decision. Our risk signature is composing of seven lncRNAs: *LINC00460*, *AL139351.1*, *AC156455.1*, *AL035446.1*, *LINC02471*, *AC022509.2*, and *LINC01606*.

The functions of *LINC00460* have been clarified in multiple kinds of tumors. In esophageal squamous cell carcinoma (ESCC), *cAMP*-response element binding protein binding protein (CBP) and *EP300* up-regulates the expression of *LINC00460* through binding to the promoter of *LINC00460* and regulating its chromatin architecture. The up-regulated *LINC00460* acts as an oncogene in ESCC by regulating cell proliferation, cell cycle and apoptosis in tumor cells (32). In non-small cell lung cancer (NSCLC), *LINC00460* induces epithelia-mesenchymal transition and promotes cell migration as well as invasion in tumor cells without influence the cell proliferation (33, 34). The roles of carcinogenesis of *LINC00460* were also reported in nasopharyngeal carcinoma (35), meningioma (36), colorectal cancer (37, 38), gastric cancer (GC) (39, 40), and breast cancer (41). Moreover, *LINC00460* is reported to promote the resistance to anticarcinogens. In colorectal cancer, the up-regulation of *LINC00460* leads to oxaliplatin resistance in patients with *TP53* mutations (42). In NSCLC, *LINC00460* promotes the resistance to gefitinib resistance through sponging *miR-769-5p*. The role of *LINC00460* has never been reported in ccRCC before. Our research has found its potential function of regulating cell cycle, DNA replication, mismatch repair, and drug resistance in ccRCC. *LINC02471* has been reported as a risk factor in thyroid carcinoma. The knockdown of *LINC02471* increases the expression of *miR-375*, which would inhibit the proliferation and invasion and promote the apoptosis of tumor cells (43). The function of *LINC01606* has been reported in GC. The up-regulation of *LINC01606* in GC leads to activation of *Wnt*/β-catenin and promotes the invasion and metastasis of the tumor (44). The functions of *AC022509.2*, *AL139351.1*, *AC156455.1*, and *AL035446.1* have never been reported in tumors. Our research proved their relationships with the OS of ccRCC patients and partly inferred their functions in ccRCC with enrichment analysis. The roles of these lncRNAs in tumorigenesis of ccRCC need to be further explored in experimental studies.

Risk signature has been broadly used in prognosis prediction in tumors. Several risk signatures, including lncRNAs based risk signatures, have been developed for ccRCC and appeared satisfactory predictive effectiveness (45–48). However, the roles of GID-lncRNAs were rarely mentioned in ccRCC. Our research combined the somatic mutation profiles and transcriptome profiles of ccRCC for the first time to identify GI-related lncRNAs and develop a risk signature for prognosis predicting and treatment deciding accordingly. Following a series of analyses, we have developed the risk signature successfully, validated its effectiveness in prognosis prediction and medication guidance, and partly clarified the functions of the lncRNAs participating in the risk signature. Our study provides novel insight to the influence of GI in ccRCC.

Nonetheless, we have to acknowledge some limitations of our study. Firstly, the effectiveness of our risk signature has only been validated in the TCGA cohort as we failed to obtain a valid external testing dataset containing the expression levels of all lncRNAs composing the risk signature. We would like to collect ccRCC samples by ourselves in the following clinical work and further validate the credibility of our risk signature in the future. Secondly, the roles of the seven lncRNAs in the risk signature have not been clearly clarified. More experimental and clinical studies are needed in the future.

## Conclusions

The present study integrated the somatic mutation profiles and the transcriptome profiles of ccRCC for the first time and developed a genomic instability derived lncRNAs based risk signature for prognosis predicting and therapeutic scheme deciding. We validated the reliability of the risk signature and partly interpreted the roles of the seven lncRNAs included in the risk signature in ccRCC. Our study provides novel insights into the relationships between genomic instability and lncRNAs and the roles of genomic instability derived lncRNAs in ccRCC.

## Data Availability Statement

Publicly available datasets were analyzed in this study. This data can be found here: https://portal.gdc.cancer.gov/.

## Author Contributions

HY and XX contributed equally to this work. HY and XX conceived, designed, and conducted the study, as well as wrote the manuscript. All authors reviewed the manuscript and participated in the language modification. All authors contributed to the article and approved the submitted version.

## Conflict of Interest

The authors declare that the research was conducted in the absence of any commercial or financial relationships that could be construed as a potential conflict of interest.
